# Prediction of treatment outcome in clinical trials under a personalized medicine perspective

**DOI:** 10.1038/s41598-022-07801-4

**Published:** 2022-03-08

**Authors:** Paola Berchialla, Corrado Lanera, Veronica Sciannameo, Dario Gregori, Ileana Baldi

**Affiliations:** 1 Center of Biostatistics, Epidemiology and Public Health, Department of Clinical and Biological Sciences, University of Torino, Regione Gonzole 10, Orbassano, 10043 Turin, Italy; 2 Unit of Biostatistics, Epidemiology and Public Health, Department of Cardiac, Thoracic, Vascular Sciences and Public Health, University of Padova, Padova, Italy

**Keywords:** Randomized controlled trials, Biostatistics

## Abstract

A central problem in most data-driven personalized medicine scenarios is the estimation of heterogeneous treatment effects to stratify individuals into subpopulations that differ in their susceptibility to a particular disease or response to a specific treatment. In this work, with an illustrative example on type 2 diabetes we showed how the increasing ability to access and analyzed open data from randomized clinical trials (RCTs) allows to build Machine Learning applications in a framework of personalized medicine. An ensemble machine learning predictive model is first developed and then applied to estimate the expected treatment response according to the medication that would be prescribed. Machine learning is quickly becoming indispensable to bridge science and clinical practice, but it is not sufficient on its own. A collaborative effort is requested to clinicians, statisticians, and computer scientists to strengthen tools built on machine learning to take advantage of this evidence flow.

## Introduction

Randomized clinical trials (RCTs) are the study design of choice for drawing inferences about a potential causal relationship between treatment and patient outcomes^1^. However, in the clinical practice settings, personalized or precision medicine, tailored to individual patient’s characteristics, has questioned the value of average treatment effects estimated by RCTs when dealing with target populations that usually differ from those represented by RCT participants^2^.

In this regard, some of the shortcomings in conventional medicine, which personalized medicine seeks to address, include differences in treatment response and incidence of adverse reactions based on individual variations. In personalized medicine, the focus is on identifying which interventions will be effective for patients based on their genetic, environmental, and lifestyle factors. Carrying out heterogeneous treatment effect analysis^3^, researchers can stratify individuals into subpopulations that differ in their susceptibility to a particular disease or their response to a specific treatment and identify who benefit most from a particular treatment instead of relying on an average effect estimated on a general population.

The need for new tools to store, manage, and analyze big data has been identified as a critical factor in personalized medicine’s implementation and success^4^. Much progress is expected from the digitalization of clinical research and reuse of de-identified open data for secondary research purposes in a wide area of health applications^5^. Given an appropriate data quality level, data-intensive research using machine learning (ML) could be a turning point for biomedical research and personalized medicine^6^.

ML is an interdisciplinary field aimed at developing models with maximal predictive accuracy, and it is highly tied to the concept of personalized medicine^7,8^. ML algorithms' distinctive key is their capability to improve their predictive performance through experience^9^. Typical applications include searching for novel patterns^10^, making a diagnosis or outcome prediction^11^, and optimizing treatment decisions^12^. For these reasons, ML is increasingly applied to clinical studies, and it represents a new approach towards conducting medical research and developing ways to predict individual outcomes^13^.

One of the biggest promises of ML is to assist medical decision-making in many domains. A core problem that arises in most data-driven personalized medicine scenarios is estimating of heterogeneous treatment effects. It occurs in RCTs where the goal is to estimate the effect of a treatment on the clinical response as a function of patient characteristics.

Here we discuss both the opportunities and challenges, namely the validation of findings, posed to personalized medicine by the increasing ability to access and analyze open data from RCTs.

This paper aims to investigate ML predictive capabilities in clinical trials to find evidence of patients-specific treatment effects (heterogeneity) and target responsive subgroups of patients. The paper is organized as follows: Materials and Methods section briefly introduces the illustrative example and presents the ensemble model of supervised ML algorithms. The strategy to investigate the model’s predictive capabilities to find evidence of heterogeneous treatment effect and identify the best responsive patients is also presented.

## Methods

### Illustrative example

A common concern to applying RCT-based estimates to a target population is that many clinical features that differ between the RCT study and target population modify the treatment effect. Our illustrative example is a sub-analysis of a large RCT to examine whether DPP-4 inhibitors provide better glycemic control to conventional therapy in patients with type 2 diabetes. In this example, we exploited ML capabilities to identify systematic variation in treatment outcome, separate it from the variation due to the sampling error and target responsive subgroups of patients.

To conduct such a heterogeneous treatment analysis, we focused on the PROLOGUE RCT^14^. The PROLOGUE study is among the largest trials investigating whether DPP-4 inhibitors provide cardiovascular protective effects to patients with type 2 diabetes by slowing carotid stiffness progression associated with conventional diabetes treatment.

The study participants were either allocated to add-on DPP-4 inhibitor (Sitagliptin) treatment or continue therapy with conventional anti-diabetic agents. The primary endpoint was the arterial stiffness’s annual changes, which did not significantly differ between the two groups. However, the study showed that the decrease in Glycated Haemoglobin (HbA1c) in patients treated with Sitagliptin was superior to conventional therapy, proving a better glycemic control. As a sub-analysis of the PROLOGUE study, we then investigated a potential heterogeneous Sitagliptin effect on improving HbA1c.

ML algorithms need to learn the statistical dependencies between clinical features and patients’ treatment outcomes; therefore, we focused on the SAIS1 RCT^15^ to train the outcome prediction model. The SAIS1 is a multicenter, prospective randomized parallel-group study comparing the effect of two DPP-4 inhibitors (Sitagliptin and Glimepiride) on endothelial functionality in patients with type 2 diabetes.

Both the SAIS1 and the PROLOGUE RCTs have collected a common subset of patient measures and share the same inclusion and exclusion criteria (see Supplementary Table S1), making them suitable for our investigation’s purpose.

Thus, to evaluate the ML predictive capabilities to find evidence of heterogeneous treatment in an RCT setting, our primary strategy was to train an ML model to learn statistical dependencies between the reduction of HbA1c at 6 months (outcome) and clinical characteristics of patients in the treatment arm (i.e., Sitagliptin) of the SAIS1 RCT and assess its accuracy. Then, we used the predictive outcome model developed to compute for each patient in the PROLOGUE study the probability of lowering HbA1c. By selecting different probability values to be responders, we identified subgroups of best responsive patients on whom we estimated the Sitagliptin effect, assessing the presence of a heterogeneous treatment effect.

All the methods were performed following relevant guidelines and regulations.

### Machine learning approach

No single ML algorithm is universally the best-performing technique for all datasets^16^. We adopted a weighted combination, also known as an ensemble of algorithms. Ensemble algorithms have proved to give accurate estimates across many different fields. The ensemble approach broadens from one-to-many potential learners, each building on its assumptions. In fact, despite their flexibility, ML algorithms performance on a given problem depends on how well their assumptions fit with the data.

We build on the ensemble algorithm called Super Learner (SL), which uses a cross-validated measure of prediction performance to weight each algorithm's contribution to the final prediction. There is a need for SL to include relevant predictors as part of any predictive model. The ensemble approach is a weighted average that allows multiple models to contribute to a prediction in proportion to their trust or estimated performance.

Building an SL requires defining a set of algorithms or learners $({\Psi }_{1}.\dots {\Psi }_{L}$) appropriate for the classification task. Their classification error is assessed using fivefold cross-validation. All the learners are trained on the same 4-folds, and their out of fold predictions are retained. Then for each algorithm, the error is estimated: the difference between each observation and prediction in the out-of-fold set is averaged. In other words, the mean squared error between the observed outcomes in the out-of-fold set and the predicted ones based on the algorithms fit on the training set is estimated.

Then the estimated error is averaged across the out-of-folds to get the cross-validated prediction error for each algorithm. Finally, to compute the contribution of each candidate algorithm to the final Super Learner prediction, non-negative least squares is used to regress the actual outcome against the cross-validated. The final SL obtained is theoretically proved to be asymptotically as good as the best candidate between learners^17^. Then, the ensemble model obtained can be used to make predictions on new data.

For a sample size of about 50–70 patients, it is suggested to use 5-folds cross-validation to assess the accuracy error of the SL^18^.

A Statistical Model (SM) is a family of probability distributions, which embody the data generating mechanism process, indexed by a set of parameters^19^. ML is taken to mean an algorithmic approach that does not use traditional identified statistical parameters and for which a preconceived structure is not imposed on the relationships between predictors and outcomes^20^.

In the following, a short description of the statistical models (SMs) and ML algorithms used as base learners is provided:

*Gradient Boosting Machine* (GBM), a tree-based ML model involving a recursive addition to the initial learning from the residuals, was applied. It fits a tree-based model on the residuals using the specified list of variables at hand and explains the variance in the residuals. The total number of trees set for the model building was 500 with interaction depth as 5, and the learning weight of iteration was 0.1^21^.

*Generalized Linear Model* (GLM) with elastic net regularization is a regression method, and as such, an SM that linearly combines the L1 and L2 penalties of the lasso and ridge methods applied in synergy with a link function a variance function to overcome linear model limitation (such as the constant variability among the mean and the normality of the data)^22^.

*Multivariate adaptive regression splines* is an SM that uses non-parametric regression method to model nonlinearities and interactions between covariates^23^.

*Random Forest* (RF) is a typical ML technique, which works by recursively creating decision trees. It selects a subset of available features and recursively partitions the data in the regression space until the subspace variation is small enough. RF is a greedy technique, and as a result, it does not necessarily converge to the optimal global solution. Bagging methods, the ensemble of locally optimal trees, provide a solution to avoid such indecisive convergence. The ensemble of such trees is known as a forest^24,25^.

*Classification and Regression Trees* (CART) are ML methods for constructing prediction models obtained by recursively partitioning the data space and fitting a simple prediction model within each partition. As a result, the partitioning can be represented graphically as a decision tree^26^.

*Bayesian Additive Regression Trees (BART)* is an ML ensemble method that relays on a prior-regularized sum-of-tree model, which prevents individual fitted trees to be dominant, powered by an iterative Bayesian back-fitting MCMC algorithm based on a likelihood built on the data in the terminal nodes. Sums of regression trees have a remarkable ability in capturing interactions, non-linearities, and additive effects^27^.

*Support-Vector Machine (SVM)* it is an ML method based on projecting of the feature space in a higher-enough dimensional space (possibly of infinite dimension). The classes are linearly separable by a hyper-plane. SVMs are among the most widely used ML techniques for classification since they ensure low computational complexity^28^.

### Statistical analysis

We set as outcome an improvement of at least − 0.5% in HbA1c, obtaining a dichotomized outcome, according to guidelines that consider a difference of 0.5% (5.5 mmol/mol) to be clinically significant^29^.

As predicting covariates, we used the common clinical patients’ characteristics collected by the two RCTs: age (years), gender (female, male), Body Mass Index (BMI, kg/cm2), Systolic Blood Pressure (SBP, mmHg), Diastolic Blood Pressure (DBP, mmHg), hypertension (SBP >  = 130 mmHg OR DBP >  = 80 mmHg), Low-Density Lipoprotein (LDL, mg/dl), High-Density Lipoprotein (HDL, mg/dl), HbA1c (%), Fasting Plasma Glucose (FPG, mmol/l), dyslipidemia (LDL >  = 130 mg/dl OR HDL < 35 mg/dl OR triglyceride >  = 150 mg/dl OR total cholesterol (= LDL + HDL + (Triglyceride/5)) >  = 200 mg/dl), adiponectin (mg/l).

Five out of 48 patients in the Sitagliptin arm of the SAIS1 study for whom HbA1c measures were not collected during the follow-up were excluded from the analysis.

To handle missing values on covariates in the PROLOGUE study, as imputation strategy, we have used a Multivariate Imputations by Chained Equations (MICE) approach^30^, using random forests^24,25^ as elementary imputation method. We have performed the imputation with a monotone visit sequence, i.e. the variables are sorted by the increasing amount of missingness to impute the data during each step through the data^30^.

The learners used were considered on the set of variables and on the subsets selected by a random forest. Supplementary Table S2 reports the variables involved in the training of each base learner. Overall, 26 (i.e. 13 × 2) different algorithms were evaluated to build on the SL. In Table 1, the learners employed and their implementation is listed. They were combined in the SL using the Non-Negative Least Squares algorithm as a meta learner, i.e. the weights they contribute are estimated to minimize the squared prediction error. The procedure for weights computation starts assigning each model a weight equal to 1/n, where n is the number of learners. Next, it evaluates the prediction performance, modifying the weight accordingly. Since the prediction performance is assessed using a fivefold CV procedure, on a sample size of 43 patients each validation set comprises 8 or 9 patients. Thus, the AUC varies with steps of 0.111 or 0.125. Given that the AUC of the learners at the first step (weights equal to 1/n) are similar, we can argue that their performance on the validation sets at each CV step remain very similar. So, it is reasonable that also the final combination is made of equal weights.

**Table 1:** Base learners and super learner (SL) training performance.

Algorithms	R implementation	Risk (1-AUC)	Weight
BART	bartMachine	0.374	0.012
BART on variables selected by RF	bartMachine_screen.randomForest	0.456	0.012
Random Forest	Caret	0.452	0.012
Random Forest on variables selected by RF	Caret_screen.randomForest	0.468	0.012
Recursive Partitioning and Regression Trees	Rpart	0.592	0.012
Recursive Partitioning and Regression Trees on variables selected by RF	Rpart_screen.randomForest	0.592	0.012
Bagging	ipredbagg	0.610	0.012
Bagging on variables selected by RF	ipredbagg_screen.randomForest	0.582	0.012
Kernel SVM	KSVM	0.477	0.012
Kernel SVM on variables selected by RF	KSVM_screen.randomForest	0.646	0.012
Elastic Net regularized GLM on variables selected by RF	GLMNET_screen.randomForest	0.549	0.012
Logistic model	GLM	0.379	0.012
Logistic model on variables selected by RF	GLM_screen.randomForest	0.428	0.012
Not adjusted logistic model	GLM	0.546	0.012
Not adjusted logistic model on variables selected by RF	GLM_screen.randomForest	0.546	0.012
Multivariate adaptive polynomial regression splines	Polymars	0.592	0.012
Multivariate adaptive polynomial regression splines on variables selected by RF	Polymars_screen.randomForest	0.546	0.012
Super Learner		0.079	

Hyperparameter tuning of the SL was conducted by a fivefold cross-validation process for which each fold was balanced to maintain the same ratio of the overall dataset in each training and validation sample. The overall performance was measured by the Area Under the Receiver Operating Characteristics Curve (AUC-ROC).

We use the outcome prediction model developed on the SAIS1 study to assign each patient the probability of being a responder (i.e., getting a reduction at 12 months of HbA1c at least of 0.5%) to each patient in the (imputed) PROLOGUE dataset.

Using distinct probability thresholds to predict PROLOGUE patients as responders to the therapy (patients successfully achieving the reduction of $\mathrm{\Delta HbA}1\mathrm{c }< -0.5\mathrm{\% })$ we sub-set the PROLOGUE patients into nested groups. The cut-off values used for the AUC-ROC computation on the SAIS1 study were selected as probability thresholds to define nested sub-groups of responsive patients in the PROLOGUE study.

For each subgroup of patients, an independent GLM was used to estimate the effect of Sitagliptin on the variation at 12 months of HbA1c (on the continuous scale) adjusted by the characteristics used in the PROLOGUE RCT to balance the allocation of patients into the two arms, i.e., treatment of diabetes mellitus before randomization (pharmacological or not), use of statin, age, gender, SBP at the office, baseline HbA1c and Maximum Common Carotid Intimal Medial Thickness.

R software^31^, version 4.0.3, was used for the analysis.

## Results

The PROLOGUE study initially involved and analyzed data on n = 385 patients with type 2 diabetes, at least 30 years of age, and levels of HbA1c between 6.2 and 9.4% at the baseline. Patients were allocated on conventional therapy (n = 193), and on Sitagliptin (n = 192). Follow-up information was collected at 12 and 24 months (data available at https://datadryad.org/resource/doi:10.5061/dryad.qt743/2) (Table 2).

**Table 2:** SAIS1 and PROLOGUE patients’ characteristics.

	SAIS1		PROLOGUE	
	Sitagliptin (N = 43)	Conventional (N = 192)	Sitagliptin (N = 193)	Combined (N = 385)
Age	55.50/61.00/67.00	64.00/70.00/76.00	64.00/70.00/75.25	64.00/70.00/76.00
Male	67% (29)	69% (134)	66% (126)	68% (260)
BMI	22.85/25.20/29.15	22.32/24.39/27.05	22.63/24.93/27.42	22.42/24.62/27.08
Hypertension	60% (26)	52% (100)	56% (108)	54% (208)
Dyslipidemia	53% (23)	49% (94)	41% (79)	45% (173)
Adiponectin	1.40/2.20/3.05	1.93/3.64/6.24	2.03/3.53/5.22	1.98/3.53/5.67
SBP	118/129/139	117.00/128.00/140.00	120.00/130.00/138.25	118.00/129.00/140.00
DBP	71.5/78/84.5	64.00/72.00/80.00	64.75/73.00/80.00	64.00/72.00/80.00
HbA1c (%)	7.1/7.4/7.8	6.6/6.9/7.2	6.5/6.8/7.2	6.5/6.8/7.2
FPG	7.3/7.9/8.8	6.16/6.94/8.21	6.15/6.94/8.38	6.16/6.94/8.27
LDL	88.5/100/117.5	77.40/89.40/112.20	77.53/94.90/109.30	77.40/92.40/111.00
$\Delta Hba1c$	− 1.15/− 0.7/− 0.3	− 0.3/− 0.5/0.00	− 0.7/− 0.35/− 0.2	− 0.6/− 0.3/− 0.10

Continuous variables are reported as 1st quartile/median/3rd quartile; categorical variables are reported as frequencies and percentage.

Body Mass Index (BMI, kg/cm^2^); hypertension (SBP >  = 130 mmHg OR DBP >  = 80 mmHg); dyslipidemia (LDL >  = 130 mg/dl OR HDL < 35 mg/dl OR triglyceride >  = 150 mg/dl OR total cholesterol (= LDL + HDL + (Triglyceride/5)) >  = 200 mg/dl); adiponectin (mg/l), Systolic Blood Pressure (SBP, mmHg); Diastolic Blood Pressure (DBP, mmHg) HbA1c (%), Fasting Plasma Glucose (FPG, mmol/l); Low-Density Lipoprotein (LDL, mg/dl); reduction of glycated haemoglobin (HbA1c) at 6 months for SAIS1 and at12 months for PROLOGUE ($\Delta Hba$ 1c).

The SAIS1 study involved n = 103 patients with type 2 diabetes, between 20 and 75 years of age, and levels of HbA1c between 6.9% and 8.4% at the baseline (data available at https://doi.org/10.1371/journal.pone.0164255.s004), who were allocated to receive Glimepiride (n = 55) or Sitagliptin (n = 48). Follow-up information was collected once, at 6 months (data available at https://doi.org/10.1371/journal.pone.0164255.s005) (Table 2).

Outcome results of both the SAIS1 and the PROLOGUE study are reported in Table 3.

**Table 3:** Outcome results in SAIS1 and PROLOGUE Study.

SAIS1 Study	Glimepiride (N = 49)	Sitagliptin (N = 43)	p-value
$\Delta$ HbA1c ≤ − 0.5%	69% (34)	70% (30)	0.969
$\Delta HbA1c$	− 0.90/− 0.70/− 0.40	− 1.150/− 0.700/− 0.30	0.316

PROLOGUE Study	Conventional (N = 193)	Sitagliptin (N = 192)	
$\Delta$ HbA1c ≤ − 0.5%	30% (57)	42% (81)	0.010
$\Delta HbA1c$	− 0.50/− 0.30/0.00	− 0.70/− 0.35/− 0.20	0.037

Best responders in PROLOGUE Study	Conventional (N = 122)	Sitagliptin (N = 131)	
$\Delta HbA1c$	− 0.5/− 0.2/0.00	− 0.7/− 0.4/− 0.2	0.013

$\Delta HbA1c$: reduction of glycated haemoglobin (HbA1c) at 6 months for SAIS1 and at 12 months for PROLOGUE.

The performance of the outcome predictive models developed on SAIS1 study patients was measured by the cross-validated AUC-ROC, which resulted equal to 92.05%. In Table 1, each learner’s error rate and weight, with which it contributes to the ensemble SL, are reported.

**Figure 1:**
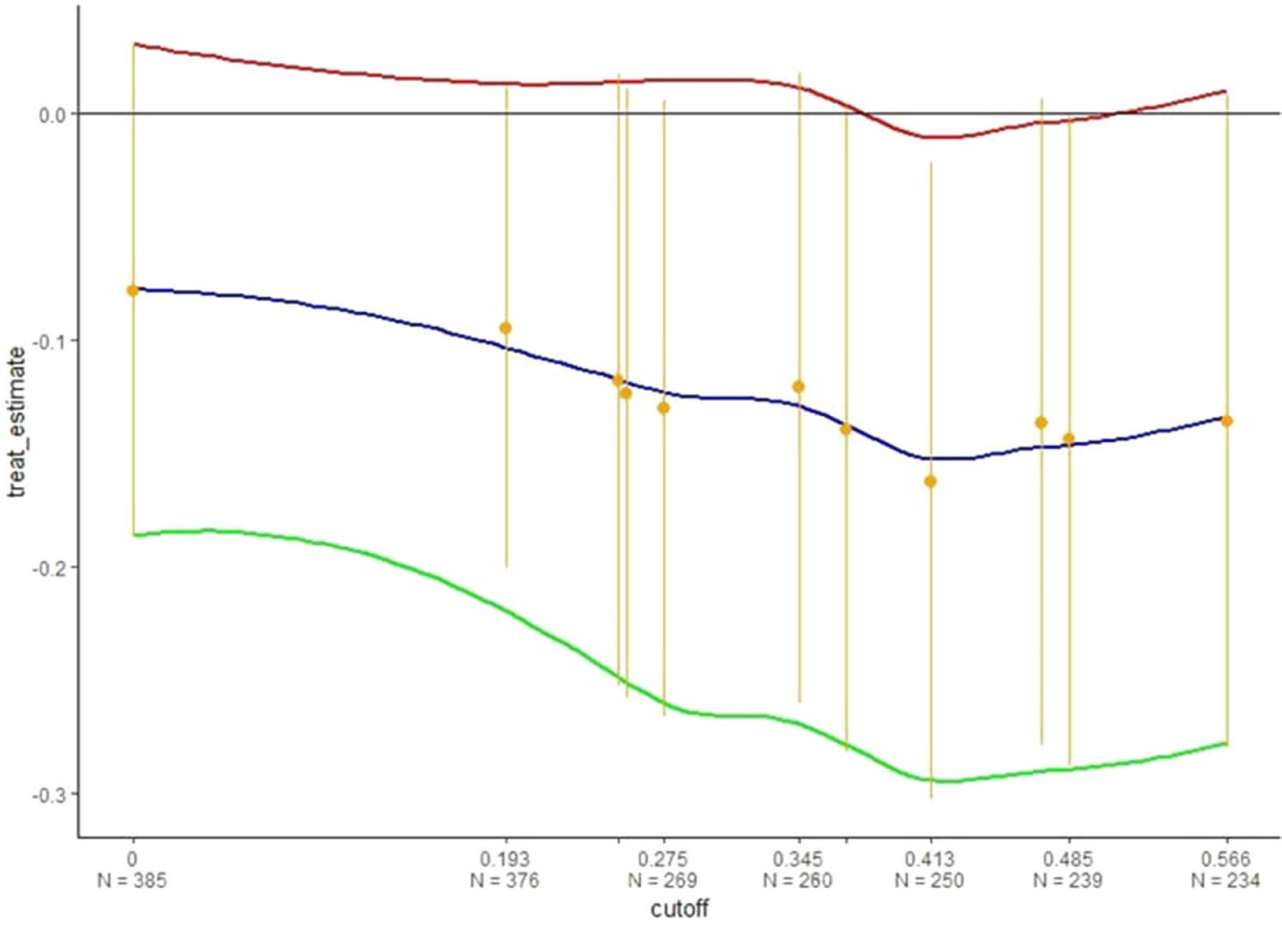
Treatment effect and 95%Confidence Intervals achieved by varying the probability (cut-off level) that defines the best responsive patients to DPP-4 inhibitor Sitagliptin. Below the cut-off levels, the number of responsive patients is reported.

The cut-off values used for the AUC-ROC computation on the SAIS1 study were selected as probability thresholds to define nested sub-groups of responsive patients. Figure 1 shows the treatment effect estimated for different sub-groups of responders selected by varying the probability value determining the responsive patients to Sitagliptin. At value 0, the estimated treatment effect is on all 385 patients. Overall, 376 out of 385 patients have a probability of getting a reduction of Ha1bc ($\Delta$ HbA1c ≤ − 0.5%) of at least 19.3%. Then, 259 out of 385 patients have a probability of achieving $\Delta$ HbA1c ≤ − 0.5% of at least 27.5%. The best treatment effect is achieved in a sub-group of 253 patients selected at the probability value of 41.3%.

On this sub-group of patients (Best responders in PROLOGUE Study in Table 3), the median reduction of glycated haemoglobin at 12 months Δ_0–12_ HbA1c is − 0.2 (IQR: − 0.5;0) among the 122 best responsive patients in the conventional group and − 0.4 (IQR: − 07; − 0.2) among the 131 best responsive patients in the Sitagplitn group. The difference among arms is still statistically significant, p = 0.013. Moreover, the two arms of best responders are still balanced for baseline characteristics (data not shown).

**Figure 2:**
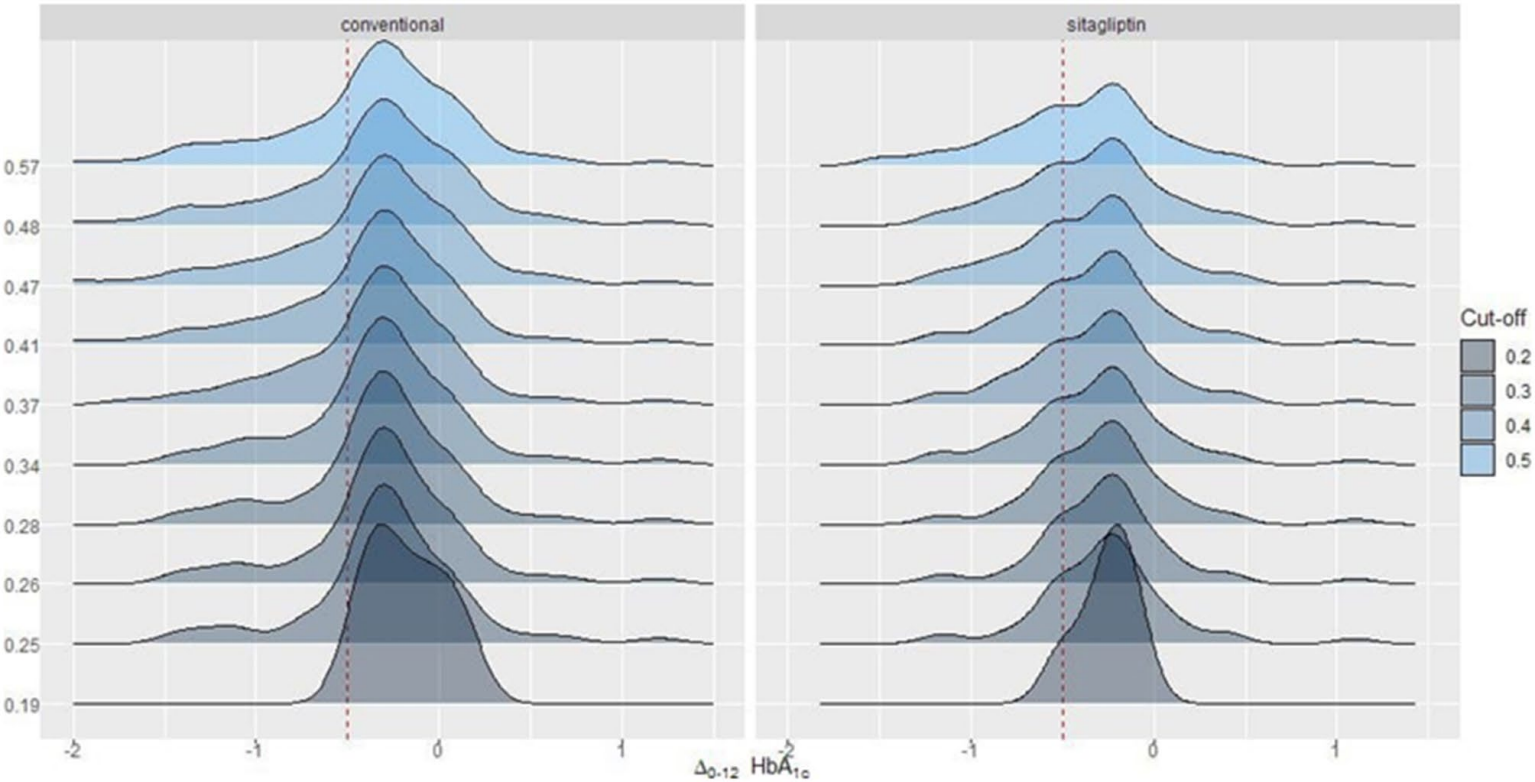
Distribution of HbA1c difference at 12 months (Δ_0–12_ HbA1c) among patients targeted as best responsive in both conventional and Sitagliptin arms according to increasing levels (from bottom to top) predictive probability of lowering HbA1c more than 0.5% (red-dashed line). At level 0, distributions of Δ_0–12_ HbA1c are based on the entire RCT sample.

Figure 2 shows the distribution of the HbA1c arm among those who were retained in both conventional and Sitagliptin arms at different pre-specified levels of the probability of being a responder. The treatment effect is assessed at each subset of patients retained, and it is not constant across these different patient subpopulations. This effect can be attributed to the heterogeneity of treatment effect and suggest an interaction between treatment and patient characteristics.

## Discussion

Precision medicine aims to target the proper treatment to suitable patients. As such, identification of non-random variation in the direction or magnitude of a treatment effect for subgroups within a population is the basis of precision medicine. In clinical trials, individual response to treatment can also be used to improve patients' enrollment and identify patient sub-populations.

In a recent scoping review^3^, Rekkas and colleagues identified many methodological approaches for assessing the heterogeneity of treatment effects in RCTs developed in the past 20 years. They grouped predictive models into three broad categories (i.e., risk-based, treatment effect modelling and optimal treatment regimen methods) depending on whether and how they incorporated prognostic variables and relative treatment effect modifiers.

Senn et al.^32^ showed how to estimate the component of variation corresponding to a patient by treatment interaction and investigate the possibility of individual response to treatment from a replicate cross over study.

In the present work, we illustrated an ML framework to carry out a heterogeneous treatment analysis in the context of RCTs. We take advantage of publicly available data upon publication of two clinical trials (SAIS1 and PROLOGUE studies) that share inclusion and exclusion criteria and a set of common clinical patients’ features. One of them (SAIS1 study^15^) was used to train an outcome prediction model, which was subsequently applied to the patients enrolled in the second trial (PROLOGUE study^14^).

Whereas in Senn et al.^32^, the patient-by-treatment interaction turned out to be unimportant, in our analysis, the heterogeneous treatment analysis made it possible to identify a subgroup of best responders to the treatment. This illustrates the potential applicability of ML in addressing the issue of finding evidence of individual patient response to treatment.

As clinical research is getting increasingly patient-driven, opportunities to deploy artificial intelligence, especially ML, are overgrowing in the perspective of precision medicine. In the last decade, cutting-edge ML techniques have advanced to a degree of maturity that allows them to be employed under real-world conditions to assist decision-making in medical and healthcare settings^33^. Their added value must be demonstrated through external validation and benchmarked in an explainable, ethical, repeatable, and scalable way.

To avoid the problem of ML, that there is no one best-performing algorithm for all situations and thus to avoid building on several models and choose the out-performing one, we used an ensemble approach called Super Learner. SL has the advantage of weighting more algorithms that contribute more accurately to the final estimate, without forcing to choose an individual model/algorithm.

Our framework focused on openly and publicly available clinical trials data. As medical research is becoming more patient-driven, the need for broader access to clinical trial data is getting more urgent. Even if still not widely adopted, open data policies^34^ have renewed the focus on sharing clinical trial data in peer-reviewed scientific journals, with profound implications in clinical practice and research.

Following this approach, ML can be adopted into the clinical trial ecosystem step-by-step, shifting the focus from the framework of clinical trials to personalized medicine^8,13^. RCTs generate immense operational data but consolidating all data—whatever the source—on a shared analytics platform, supported by open data standards, can foster collaboration and knowledge. Furthermore, incorporating a self-learning system designed to improve predictions can proactively deliver reliable analytics insights to users.

## Supplementary Information

Supplementary Table S1.

Supplementary Table S2.

## Data Availability

Publicly available datasets were analyzed in this study. Data can be found here: [https://datadryad.org/resource/doi:10.5061/dryad.qt743/2] and [https://doi.org/10.1371/journal.pone.0164255.s004].
