# Supplementary material for: Prediction of treatment outcome in clinical trials under a personalized medicine perspective
Source: Sci Rep. 2022 Mar 8;12:4115. doi: 10.1038/s41598-022-07801-4 (PMC8904517; doi:10.1038/s41598-022-07801-4)
Supplement: Supplementary file 1 — Supplementary Table S1. [file 41598_2022_7801_MOESM1_ESM.docx]

**Table S1**. Key elements and common features of SAIS1 and PROLOGUE protocols

| The Effect of Sitagliptin on Carotid Artery Atherosclerosis in Type 2 Diabetes: The PROLOGUE Randomized Controlled Trial | A Randomized Controlled Trial Comparing the Effects of Sitagliptin and Glimepiride on Endothelial Function and Metabolic Parameters: Sapporo Athero-Incretin Study 1 (SAIS1) |
| --- | --- |
| Data access:  <https://datadryad.org/resource/doi:10.5061/dryad.qt743/2> | Data access:  <https://doi.org/10.1371/journal.pone.0164255.s004> (baseline)  <https://doi.org/10.1371/journal.pone.0164255.s005> (follow-up) |
| N= 463 | N=103 |
| Time window: November 2010 and September 2012 | Time window: March 2011 to 30 September 2013 |
| Inclusion criteria: | Inclusion criteria: |
| - age ≥ 30 y | - age >20 to 75 years |
| - HbA1c ≥ 6.2% and < 9.4% despite treatment with diet, exercise, and/or conventional antidiabetic agents (Sulfonylureas, Biguanides, α-Glucosidase inhibitors, Thiazolidinedione). | - HbA1c > 6.9 and < 8.4% despite treatment with diet, exercise, and/or metformin (biguanide antidiabetic agent) |
|  | - adequate control of blood pressure and dyslipidemia |
| Exclusion criteria: | Exclusion criteria: |
| - type 1 diabetes mellitus |  |
| - undergoing insulin treatment | - undergoing insulin treatment; |
| - administration of DPP-4 inhibitors and/or GLP-1 analogues before randomization | - history of hypersensitivity to insulin or GLP-1 receptor agonists |
| - heart failure with New York Heart Association functional class III or IV | - patients who take more than four antihypertensive medications; heart failure |
| - a history of diabetic ketoacidosis or diabetic coma within the 6 mo prior to randomization |  |
| - a history of myocardial infarction, angina pectoris, percutaneous transluminal coronary angioplasty, or bypass surgery | - atherosclerotic diseases (angina, myocardial infarction, cerebral infarction and peripheral arterial disease) |
| - a history of cerebral infarction, cerebral hemorrhage, subarachnoid hemorrhage, or transient ischemic attack within the 3 mo prior to randomization | - atherosclerotic diseases (angina, myocardial infarction, cerebral infarction and peripheral arterial disease) |
| - serious renal dysfunction (estimated glomerular filtration rate < 30 ml/min/1.73 m^2^ or dialysis) | - persistent elevation of their serum transaminase levels or had renal dysfunction |
| - pregnancy or possible pregnancy | - pregnancy |
| - lack of informed consent | - lack of informed consent |
| - judgment of the investigator that an individual is ineligible for inclusion in the study | - judgment of the investigator that an individual is ineligible for inclusion in the study |
| Treatment: randomly assigned (ratio 1:1) either to receive | Treatment: randomly assigned (ratio 1:1) either to receive |
| - conventional therapy plus sitagliptin (DPP-4 inhibitor)(sitagliptin group) | - once daily sitagliptin (50–100 mg/day) + (diet, exercise) |
| - only conventional therapy (diet, exercise, and/or antidiabetic agents, except for DPP-4 inhibitors, GLP-1 analogues, and insulin; conventional therapy group). | - glimepiride (0.5–2.0 mg/day) + (diet, exercise) |
| Randomization: modified minimization method with a biased-coin assignment balancing on age (<65 or ≥65 y), sex, use of statins, use of antidiabetic agents (nonpharmacological or pharmacological), HbA1c (<7.0% or ≥7.0%), office systolic blood pressure (<135 or ≥135 mm Hg), and maximum IMT (<1.0 or ≥1.0 mm). | Randomization balancing age, body mass index and results of FMD |
| Follow-up: 2 yrs | Follow-up: 24 weeks of treatment |
| Primary Endpoint at 24 months:   - % change in mean CCA IMT | Primary Endpoint at 24 weeks (6 months):   - the extent of change in FMD |
| Secondary endpoints at 12 and 24 months:   - the mean and maximum IMT values and changes at the CCA, bulb, and ICA (except for the primary endpoint); - plaque area and plaque gray scale median; - the values and changes in glycemic profiles (HbA1c, fasting glucose level, insulin concentration, 1,5-anhydroglucitol,1,4-anhydro-D-glucitol, HOMA-β, HOMA-R); lipoprotein profiles (total cholesterol, high-density lipoprotein cholesterol, triglyceride, small dense low-density lipoprotein, malondialdehyde-modified low-density lipoprotein, remnant-like particle cholesterol); renal function (creatinine, cystatin C, urinary albumin/creatinine ratio, estimated glomerular filtration rate); high molecular weight adiponectin; physiological parameters (body weight, blood pressure); - adjudicated clinical events and adverse events. | Secondary endpoints at 24 weeks (6 months):   - mean changes between baseline and post-treatment of endothelial and metabolic parameters (listed in table 4: BMI, HbA1c, SBP, DBP, HDL, LDL, …) |
| Predictors:   - Age, years - Sex - Body mass index, kg/m2 - Hypertension (history of) - Dyslipidemia (history of) - Adiponectin - Myocardial infarction - Percutaneous coronary intervention - Coronary artery bypass grafting - Chronic heart failure - Arrhythmia - Stroke - Systolic blood pressure, mm Hg - Diastolic blood pressure, mm Hg - HbA1c, percent - Fasting plasma glucose (FPG), mmol/l - Low-density lipoprotein cholesterol (LDL), mmol/l - Serum creatinine, μmol/l | Predictors:   - Age, years - Gender - BMI (kg/m2) - Hypertension^1^ - Dyslipidemia - Adiponectin     - SBP (mmHg) - DBP (mmHg) - HbA1c (%) - FPG (mmol/l) - LDL (mmol/l) |

^1^ Hypertension is defined according to the 2017 American College of Cardiology/American Heart Association Guideline of systolic BP ≥130 mm Hg and/or diastolic BP ≥80 mm.

^2^ Dyslipidemia is defined according to the American Heart Associations classification corresponding to the 95th percentile in a American population as total cholesterol >5.2 mmol/L (200 mg/dl), LDL > 3.4 mmol/L (130 mg/dl), HDL <0.9 mmol/L (35 mg/dl), or triglycerides >1.7 mmol/L (150 mg/dl)
