# Supplementary material for: Prediction of treatment outcome in clinical trials under a personalized medicine perspective
Source: Sci Rep. 2022 Mar 8;12:4115. doi: 10.1038/s41598-022-07801-4 (PMC8904517; doi:10.1038/s41598-022-07801-4)
Supplement: Supplementary file 2 — Supplementary Table S2. [file 41598_2022_7801_MOESM2_ESM.docx]

**Table S2**. Features retained in each base learner after the selection provided by the Random Forest algorithm, when applied (reported “All” when the selection is not applied).

| SL.bartMachine_All | All |
| --- | --- |
| SL.bartMachine_screen.randomForest | age, gender, bmi, dyslipidemia_adj, adiponectin, sbp, dbp, hba1c, fpg, ldl |
| SL.biglasso_All | All |
| SL.biglasso_screen.randomForest | age, gender, bmi, dyslipidemia_adj, adiponectin, sbp, dbp, hba1c, fpg, ldl |
| SL.caret_All | All |
| SL.caret_screen.randomForest | age, gender, bmi, dyslipidemia_adj, adiponectin, sbp, dbp, hba1c, fpg, ldl |
| SL.caret.rpart_All | All |
| SL.caret.rpart_screen.randomForest | age, gender, bmi, dyslipidemia_adj, adiponectin, sbp, dbp, hba1c, fpg, ldl |
| SL.earth_All | All |
| SL.earth_screen.randomForest | age, gender, bmi, dyslipidemia_adj, adiponectin, sbp, dbp, hba1c, fpg, ldl |
| SL.glmnet_All | All |
| SL.glmnet_screen.randomForest | age, gender, bmi, dyslipidemia_adj, adiponectin, sbp, dbp, hba1c, fpg, ldl |
| SL.ipredbagg_All | All |
| SL.ipredbagg_screen.randomForest | age, gender, bmi, dyslipidemia_adj, adiponectin, sbp, dbp, hba1c, fpg, ldl |
| SL.ksvm_All | All |
| SL.ksvm_screen.randomForest | age, gender, bmi, dyslipidemia_adj, adiponectin, sbp, dbp, hba1c, fpg, ldl |
| SL.lm_All | All |
| SL.lm_screen.randomForest | age, gender, bmi, dyslipidemia_adj, adiponectin, sbp, dbp, hba1c, fpg, ldl |
| SL.mean_All | All |
| SL.mean_screen.randomForest | age, gender, bmi, dyslipidemia_adj, adiponectin, sbp, dbp, hba1c, fpg, ldl |
| SL.polymars_All | All |
| SL.polymars_screen.randomForest | age, gender, bmi, dyslipidemia_adj, adiponectin, sbp, dbp, hba1c, fpg, ldl |
| SL.randomForest_All | All |
| SL.randomForest_screen.randomForest | age, gender, bmi, dyslipidemia_adj, adiponectin, sbp, dbp, hba1c, fpg, ldl |
| SL.ranger_All | All |
| SL.ranger_screen.randomForest | age, gender, bmi, dyslipidemia_adj, adiponectin, sbp, dbp, hba1c, fpg, ldl |
| SL.rpart_All | All |
| SL.rpart_screen.randomForest | age, gender, bmi, dyslipidemia_adj, adiponectin, sbp, dbp, hba1c, fpg, ldl |
